# Therapy of *Mycobacterium abscessus* Infections in Solid Organ Transplant Patients

**DOI:** 10.3390/microorganisms12030596

**Published:** 2024-03-16

**Authors:** Lubna Osman, Christopher Lopez, Yoichiro Natori, Shweta Anjan, Julia Bini Viotti, Jacques Simkins

**Affiliations:** 1 Infectious Diseases, Mount Sinai Medical Center, 4300 Alton Rd, Miami Beach, FL 33140, USA; 2 Department of Medicine, Division of Infectious Diseases, University of Miami School of Medicine, 1120 NW 14th Street, Miami, FL 33019, USA

**Keywords:** *Mycobacterium abscess* complex, solid organ transplant patients, lung transplant patients, management, novel therapies

## Abstract

*Mycobacterium abscessus* complex (MABC), a rapidly growing *Mycobacterium*, is one of the most common causes of non-tuberculous mycobacteria (NTM) infections in the United States of America, and it has been associated with a wide spectrum of infections in immunocompetent and immunosuppressed individuals. Eradicating MABC is very challenging, even with prolonged combination therapies. The management of MABC infections in solid organ transplant (SOT) patients is usually complex given their net state of immunosuppression, associated comorbidities, and potential drug–drug interactions, among other things. In this manuscript, we discussed the antimicrobial management of pulmonary and extrapulmonary MABC infections. In addition, we reviewed promising novel therapies such as clofazimine, omadacycline, bedaquiline, and inhaled tigecycline that could join the existing antimicrobial armamentarium to fight this infection associated with significant morbidity and mortality. However, further studies are needed, especially among the immunocompromised host.

## 1. Introduction

Non-tuberculous mycobacteria (NTM) are common environmental organisms that can survive harsh conditions, including low pH and extreme temperatures. *Mycobacterium abscessus* complex (MABC) is one of the most common causes of NTM infections in the United States, and its prevalence is increasing [1]. It is one of the rapidly growing mycobacteria species, first reported by Moore and Freriches in 1953 [2]. Recent findings support its taxonomic status as a single species comprising three subspecies designated as *abscessus*, *bolletii*, and *massiliense* [3]. 

It is well known that MABC causes a wide spectrum of infections in both immunocompetent and immunocompromised patients [4,5] and has been associated with hospital outbreaks [6]. The incidence of MABC infection is higher among lung transplant (LT) recipients compared to other solid organ transplant (SOT) recipients, and this can be explained by the fact that LT recipients require higher immunosuppression following LT compared to other SOT recipients. LT recipients have a large number of donor-derived dendritic cells capable of T-cell activation and can be exposed to environmental antigens that could trigger graft rejection [7]. 

MABC possesses a unique species-specific repertoire of genes that confers innate resistance to multiple classes of antibiotics [8]. MABC has proven to be difficult to eradicate due to limited antimicrobial options and its ability to form biofilms, resulting in cure rates below 50% even with a long duration of effective antibiotic combination therapies [1]. MABC infection in LT recipients is associated with allograft dysfunction and increased mortality [7]. 

The approach for treating MABC infections in SOT recipients is complex and warrants unique considerations. These include the net state of immunosuppression, type of SOT, disease severity, disease location (pulmonary vs. extrapulmonary), in vitro antimicrobial susceptibilities and molecular testing for inducible macrolide resistance, potential drug–drug interactions (DDI), and ability to tolerate medications. Infections due to MABC tend to be aggressive in the post-transplant setting [9]. It has a proclivity to disseminate in times of increased immunosuppression where management can be further complicated by concomitant opportunistic infections, balancing decreasing immunosuppressive agents to combat infection while avoiding transplant rejection, which makes this topic of increasing concern in the transplant setting. A retrospective cohort study of 108 hospitalized patients with positive cultures for MABC revealed that organ transplantation itself is a risk factor for early treatment failure in MABC infections, stressing the importance of meticulous management and pursuing further studies on effective therapeutic modalities [5]. 

In this review article, we will discuss treatments for pulmonary disease with a focus on LT, management of extrapulmonary disease in SOT patients, novel therapies, and unique therapeutic considerations in the transplant population. 

## 2. Clinical Presentation and Diagnosis

The clinical presentation of MABC and other NTM in SOT recipients varies. Therefore, clinicians must have a high index of suspicion to recognize NTM infections in this vulnerable population. MABC can cause five different clinical syndromes, namely pleuropulmonary infection, skin and soft tissue infection (SSTI), osteoarticular infection, lymphadenitis, and disseminated disease, including central-line associated bloodstream infections (CLABSI). Constitutional symptoms such as fevers, night sweats, and weight loss are uncommon. Pulmonary infections typically present with cough, sputum production, and shortness of breath. SSTI, oftentimes, presents as painful erythematous or violaceous subcutaneous nodules that can sometimes ulcerate or form abscesses [9]. Contrary to other NTM, MABC is more likely to cause severe clinical manifestations in SOT recipients, given its tendency to disseminate and its multidrug-resistant feature [9]. 

Establishing the diagnosis of pulmonary MABC infection is performed by looking for the presence of clinical, radiographic, and microbiologic criteria as recommended by the joint guidelines from the American Thoracic Society (ATS)/Infectious Diseases Society of America (IDSA) [10]. There are no specific criteria for the diagnosis of extrapulmonary MABC infection. Therefore, a thorough assessment of clinical, histopathologic, and microbiologic data is often required to make the diagnosis [9]. Recovering MABC from a sterile source (e.g., blood or synovial fluid culture) provides strong evidence of invasive MABC infection [9].

## 3. Treatment of Pulmonary MABC Infection in SOT

Although pulmonary MABC infection is commonly encountered in patients with cystic fibrosis undergoing LT, it is an important consideration for any patient undergoing SOT [11]. MABC is one of the most common NTM infections in the cystic fibrosis populations, and it is suggested that MABC infection is associated with modification in some cell-mediated immunity pathways, such as increased T-cell expressing CD40L, but not IL-2 and this modification may play a part in the progression of the disease [12]. It is not surprising that a single-center study found that the median time to development of NTM infections after SOT was approximately 8 months, as the intensity of immunosuppression is often highest within the year following transplantation [9]. 

The transplant community does not have a consensus on how to manage MABC isolation among potential LT candidates. MABC isolation is considered a relative contraindication to LT by some clinicians, and some transplant centers would not perform LT in patients with MABC isolation due to concerns of increased morbidity and mortality [7].

An international expert panel in NTM recommends waiting until sputum cultures are negative for 12 months before listing patients for LT among patients with MABC lung infection who are currently undergoing NTM treatment [13]. However, this may not be practical for patients requiring urgent LT, as LT can be a lifesaving procedure, and a double lung transplant itself may reduce the burden of the MABC. LT has been carried out in patients whose respiratory cultures are still positive for MABC. In a small study performed at the University of California San Francisco from 2010 to 2018, seven patients infected with MABC received treatment prior to LT. Five out of seven were still culture-positive at the time of LT. The patients were treated for a median of 6 months post-transplant. One of the patients developed a post-operative MABC soft tissue infection that was successfully treated. The time to chronic lung allograft dysfunction (CLAD) and survival were similar to the control group [14]. In another small study, Lobo et al. reported the outcomes of 13 patients with cystic fibrosis and MABC respiratory infection who underwent LT. Three patients developed MABC-related complications, but all of them had clearance of the infection after being treated. The survival was not different in the MABC group compared to the control group [15]. In a study that included pediatric patients with cystic fibrosis and chronic MABC infection who underwent heart/lung or LT, it was found that the outcomes were worse with the subspecies *M. abscessus* subsp. *abscessus*. One patient with *M. abscessus* subsp. *bolletii*, five with *M. abscessus* subsp. *massiliense*, and seven with *M. abscessus* subsp. *abscesus* were included. One patient with *M. abscessus* subsp. *massiliense* and all seven with *M. abscessus* subsp. *abscessus* died post-lung transplant. The patient with *M. abscessus* subsp. *bolletii* and three with *M. abscessus* subsp. *massiliense* performed well after the transplant [16]. 

Bilateral LT is recommended if there is a history of MABC infection to prevent allograft infection [17]. Removing the diseased lungs would decrease the “disease burden” and facilitate MABC eradication when this surgical approach is coupled with aggressive antimicrobial treatment [7]. A native lung in a single LT could be a potential source of infection that could subsequently infect the allograft [7]. 

Not all positive cultures seen in LT recipients represent infection; thus, distinguishing active infection from colonization is essential before starting treatment [18]. MABC cultured from a sterile site with clinical syndrome is considered a true infection, and a positive culture represents colonization in a patient that does not meet the ATS/IDSA criteria for NTM disease [10].

In 2019, the American Society of Transplantation (AST) recommended for MABC lung infection starting a 3-drug therapy for at least one month until results of susceptibility testing become available, and the recommended empiric regimen was azithromycin plus two parenteral agents (amikacin, tigecycline, cefoxitin, or imipenem) [9]. The use of inhaled amikacin for pulmonary NTM lung infection has been supported, especially when patients develop nephrotoxicity from the intravenous (IV) formulation [9]. Amikacin liposomal inhaled suspension (ALIS) has a 4-fold improved uptake into macrophages compared to inhaled free amikacin, as well as the higher mean area under concentration time curve of 42- and 274-fold in lung parenchyma and macrophages, respectively, when compared with IV amikacin. The liposomal formulation penetrates extracellular mycobacteria in biofilms along airways and intracellular mycobacteria within macrophages in the pulmonary parenchyma more effectively [19]. In the United States, ALIS was approved for refractory *Mycobacterium avium intracellulare*, but it is often used in patients with MABC pulmonary infections. Data on the use of ALIS in the transplant population are limited. In a French observational study on pulmonary infection due to MABC treated with ALIS among patients with and without cystic fibrosis, those patients who underwent LT were excluded from the analysis [20]. Some of the side effects reported are QTc prolongation, hepatotoxicity and ototoxicity for azithromycin, ototoxicity and nephrotoxicity (IV formulation) for amikacin, major gastrointestinal intolerance for tigecycline, and cytopenias and hepatotoxicity for imipenem [9]. Note that some of the antibiotics used to treat MABC should be renally adjusted in the presence of renal insufficiency [9] (see Table 1).

In 2020, the joint guidelines by the ATS, IDSA, European Respiratory Society (ERS), and European Society of Clinical Microbiology and Infectious Diseases (ESCMID) recommended using at least three active drugs (based on susceptibility testing) in the initial phase of treatment for MABC lung infection. The panel recommended at least four active drugs in cases of macrolide resistance [1].

The panel of experts recommended the following antibiotics for LT cases: macrolides, amikacin (IV or inhaled), tigecycline, and imipenem for pre-transplant non-cavitary disease. Note that 75% of panelists agreed on using the same regimen for pre-transplant patients with cavitary disease. For post-transplant cases (non-cavitary and cavitary cases), they recommended macrolides, IV amikacin, and imipenem. Of note, there was no consensus on the use of inhaled amikacin [17]. The recommended duration for post-transplant MABC disease is 12 months after sputum culture conversion. Some of the experts would treat until 12 months after cavitary closure on follow-up chest computed tomography (CT) scan [17]. Adjuvant surgical resection has been performed in selected LT recipients with localized disease and adequate lung function. Resecting the infected part of the lung following initial antimicrobial treatment to decrease the “disease burden” can increase the chances of cure [7]. 

Immunosuppression reduction is also recommended if the NTM isolated is MABC with only agreement on corticosteroids but not on tacrolimus or mycophenolate mofetil [17]. Avoiding aggressive induction therapy with thymoglobulin and aiming for lower post-transplant levels of calcineurin inhibitors (CNI) have been recommended in SOT recipients with MABC disease to facilitate the treatment of the infection. However, this should be balanced against the risk of graft rejection [7]. 

## 4. Treatment of Extrapulmonary MABC Infection in SOT

Cases of extrapulmonary MABC infection have been reported among patients with different transplanted organs. In this section, we report some of the treatment data that have been published in the medical literature. 

LT patients with MABC cutaneous infection can achieve a cure with at least 6 months of combination therapy, including amikacin [21]. Single-agent treatment is not recommended as it could lead to resistance [13]. Topical amikacin cream efficacy has not been proven, but it has been used [15]. Surgical intervention, along with combination therapy, is by far the superior method in eradicating the disease [22]. Antibiotic treatment alone might not be enough due to poor penetration, and sometimes, these cutaneous infections would require several surgical drainages [7]. 

In a retrospective study, ten orthotopic heart transplant (OHT) patients and seven patients with ventricular assist device (VAD) developed extrapulmonary *M. abscessus* subsp. *abscessus* infection during a hospital outbreak linked to heater–cooler units. The most common sites of positive cultures were blood (*n* = 12), sternum/mediastinum (*n* = 8), and the VAD driveline exit site (*n* = 7). The 14 patients who were diagnosed alive received antimicrobial treatment for a median of 21 weeks, and only 8 (47%) patients survived longer than 12 weeks after diagnosis [23]. A case series of two patients with VAD infection due to MABC revealed that one patient was treated with appropriate therapy, underwent driveline un-roofing, and subsequently underwent OHT, and the other patient received appropriate therapy and had a percutaneous aspiration of the collection along the driveline and subsequently underwent VAD exchange. The cure of MABC infection was achieved in both patients [24]. 

A two-year-old patient with renal hypodysplasia on peritoneal dialysis (PD) diagnosed with MABC PD exit site infection and peritonitis was successfully treated with IV amikacin, imipenem, tigecycline, and clarithromycin for 6 months. Of note, co-management involved source control with multiple surgical debridements and PD catheter removal. Subsequently, he underwent a living donor renal transplant after 4 months without disease recurrence and had an uneventful post-transplant course with a follow-up of two years at the time the case report was published [25].

Unlike pulmonary MABC disease, extrapulmonary MABC infection tends to require a shorter duration, as it is the shortest for localized cutaneous infections [9]. Note that MABC skin infection can sometimes be a manifestation of disseminated disease [5].

The ATS/IDSA guidelines recommended treatment durations of 4 and 6 months for severe SSTI and osteomyelitis due to MABC, respectively, and at least 6–12 months from immune reconstitution for disseminated disease, including documented bloodstream infection (BSI). Treatment duration was not specified for other types of extrapulmonary MABC infection [10]. 

Obtaining an infectious disease consultation is recommended prior to the initiation of treatment for MABC infections (pulmonary and extrapulmonary) [1,9], and consulting the NTM experts at the National Jewish Health Center in Denver might be useful given how resistant and aggressive MABC can be [9]. The doses of commonly used drugs against MABC recommended by the AST [9] are listed in the Table 1.

## 5. Novel Therapies

Further studies on novel therapies are needed to determine safety, tolerability, and efficacy, considering the proclivity of MABC to have innate and acquired resistance and the poor tolerability of some of the current drugs. Specifically, inhaled formulations are of interest for pulmonary infection due to decreased systemic absorption limiting the adverse effect profile. Oral antimicrobial agents are appealing due to ease of administration and lifestyle flexibility compared with IV therapies. Classes of antibiotics with oral formulations with in vitro activity for MABC include macrolides, oxazolidinones, clofazimine, bedaquiline, and omadacycline. The novel therapy of inhaled nitric oxide will not be reviewed due to a lack of statistically significant reduction in bacterial load in sputum and no improved lung function seen in an open-label study of nine patients [26]. 

### 5.1. Clofazimine

Clofazimine is a riminophenazine dye used as an antibacterial agent for the treatment of leprosy. However, its use has been expanded to multi-drug-resistant tuberculosis and NTM infections. The utilization of clofazimine for MABC infections is uncommon, but it is often used as salvage therapy in the treatment of NTM when there is a progression of infection despite appropriate therapy, intolerance from side effects, or limited antimicrobial options based on MABC in vitro susceptibilities [27]. The clofazimine minimum inhibitory concentration (MIC) varied widely but tended to be low. Note that the chances for culture conversion are much higher in patients with an MIC value ≤ 0.25 mg/L than in patients with an MIC value > 0.5 mg/L [28]. 

The use of clofazimine in combination with other antimicrobials was associated with favorable outcomes in a cohort of LT recipients with pulmonary MABC infection. One-year survival with clearance of infection was more common among those who received clofazimine [29]. Its safety and tolerability in SOT patients can be extrapolated from a single-center study of five SOT recipients with *Mycobacterium avium* complex disease. Of the five, one discontinued clofazimine due to gastrointestinal side effects, three had skin discoloration, and none of the patients had hematologic or hepatotoxic side effects [30]. Skin discoloration and gastrointestinal side effects were the top two reasons for discontinuing this drug in a retrospective study that included 42 patients with MABC pulmonary infection [31]. In vitro data have shown that clofazimine can have a synergistic interaction when used with amikacin [32], tigecycline [33], and bedaquiline [34].

### 5.2. Omadacycline

Omadacycline is a semisynthetic tetracycline-derived aminomethylcycline antibiotic available in oral and IV formulations that has in vitro activity against NTM, including MABC. Although there are no recognized susceptibility test interpretive criteria by the Food and Drug Administration (FDA) for omadacycline for the management of mycobacterial infections, the in vitro MICs were comparable to tigecycline and eravacycline and were used as a surrogate for omadacycline susceptibility [35]. Its use in SOT patients is yet to be evaluated. A case series reviewed four cases (one pulmonary, two SSTI, and another with osteomyelitis and mycobacterial BSI), which were managed by combined regimens that included omadacycline. All four cases demonstrated clinical improvement, with a cure documented in three of the four cases. The notable side effect was nausea, resulting in the discontinuation of omadacycline after 6 months of treatment in one patient [36]. 

A multicenter, retrospective, observational case series described 12 patients who were treated with omadacycline as part of the initial antimicrobial combination therapy for MABC infection (7 pulmonary and 5 extrapulmonary) with clinical cure in 9 of the 12 patients. Extrapulmonary disease involves SSTI, osteomyelitis, and CLABSI. Clinical failure occurred in two patients with pulmonary MABC and one with localized SSTI [37]. 

In another case series, clinical cure or improvement with omadacycline-containing regimens was documented in all three patients with pulmonary MABC infection, although one patient required antiemetics to tolerate omadacycline [38]. 

Currently, there is a clinical trial assessing omadacycline monotherapy vs. placebo in MABC pulmonary infection with a phase 2, double-blinded, randomized, parallel-group, placebo-controlled multicenter study to determine safety, tolerability, and efficacy [39]. 

### 5.3. Bedaquiline

Bedaquiline is a diarylquinolone antibiotic that inhibits mycobacterial ATP synthase and is better known for its use in multidrug-resistant *Mycobacterium tuberculosis* infections as recommended by the World Health Organization [40,41]. A systematic review and meta-analysis revealed that bedaquiline-containing regimens were effective in treating NTM extrapulmonary infection but not pulmonary infection [42]. Another report revealed potential clinical and microbiologic activity of bedaquiline in patients with MABC lung disease [43]. Bedaquiline and clofazimine were used as salvage therapy after initial failed four-drug induction therapy in a child with acute myeloid leukemia and calcaneal osteomyelitis due to MABC [44]. Bedaquiline was successfully used as part of a combination therapy in two HIV patients with disseminated NTM [45]. Note that resistance to bedaquiline has been noted [42]. Bedaquiline was largely well tolerated, and the most common adverse effects were nausea and QTc prolongation [42]. 

### 5.4. Inhaled Tigecycline

Intravenous tigecycline is an important component of first-line combination therapy in MABC pulmonary and extrapulmonary infections, as recommended by current guidelines [1,9].

Systemic tigecycline can be difficult to tolerate due to adverse effects, including nausea, vomiting, pancreatitis, and hepatitis, so inhaled tigecycline was studied and has been shown to be highly effective in a dose-dependent manner in mice infected with MABC by intrapulmonary aerosol. In addition, inhaled tigecycline retained most of its activity in patients with cystic fibrosis [46]. Twice daily inhaled tigecycline (despite in vitro resistance) was used successfully as salvage therapy in combination with linezolid and clarithromycin in a 62-year-old female with bronchiectasis struggling with MABC for years. It is important to note that linezolid and clarithromycin were used in prior antimicrobial combinations without culture conversion. The main adverse effects documented were aphthous stomatitis and nausea [47]. There are no reports of use of inhaled tigecycline used in LT patients.

### 5.5. Tebipenem–Avibactam and Faropenem 

Tebipenem is an oral beta-lactam of the carbapenem class notably studied in the ADAPT-PO trial for complicated urinary tract infection, although it is yet to be FDA-approved for this indication [48]. When combined with the beta-lactamase inhibitor avibactam, it has bactericidal activity in vitro against MABC displayed in a murine model with pulmonary infection [49] and is a promising oral option with a likely more suitable side effect profile to be studied in patients with MABC, including SOT patients. The most common side effects of tebipenem are mild diarrhea and headache [48]. 

Faropenem is an oral penem that was successfully used in combination with clarithromycin for a case of MABC lung infection [50]. However, it was not approved by the FDA [51]. Therefore, its use should not be encouraged for the time being. 

### 5.6. Tedizolid

Tedizolid is an oxazolidinone-like linezolid that inhibits the 50S ribosome and has been shown to have in vitro activity against MABC alone and in combination with amikacin and clarithromycin [52,53]. It is often utilized when adverse effects of the oxazolidinone class are a concern or have already occurred with linezolid, as it may have a more tolerable side effect profile in comparison to linezolid over a 14-day treatment course [54]. Tedizolid was used successfully in combination with bedaquiline in a patient with HIV with disseminated MABC infection after developing linezolid-related myelosuppression [45]. When therapeutic options are limited and linezolid adverse effects have occurred or are feared to occur, tedizolid appears to be a viable option to consider as we await further clinical data on this agent. 

## 6. Other Drugs with Potential Activity against MABC

Another approach in the search for alternative agents for the treatment of MABC infections includes the re-purposing of currently available drugs. Egorova et al. describe that some agents, such as orlistat, the lipase inhibitor used for weight loss, and disulfuram, the aldehyde dehydrogenase inhibitor used for alcohol use disorder, have in vitro activity against MABC. Note that disulfuram displays synergistic activity with amikacin or moxifloxacin [55]. D-cycloserine, a drug used for *M. tuberculosis* infection, has in vitro activity as well and exhibits synergy with clarithromycin [56]. The anti-cystine agent cysteamine has been proven to have in vitro activity against MABC, specifically in patients with cystic fibrosis [57]. Lastly, the veterinary antibiotic thiostrepton has in vitro activity against MABC [58]. Abundant data demonstrating efficacy and safety would be required before these agents can be considered therapeutic options for MABC infections in the general and transplant population. 

### 6.1. Synergistic Effect of Rifaximin with Macrolides

Another promising novel treatment includes a combination of rifaximin with macrolides. In a zebrafish embryo MABC infection model, it was found that rifaximin was a potentiator for clarithromycin despite the presence of the *erm*(41) gene responsible for macrolide-inducible resistance. This combination was found to work synergistically and have bactericidal activity, possibly due to inhibiting the induction of the *erm*(41) gene by targeting its transcription [59]. Clinical application is needed as rifaximin is known to have poor systemic absorption, and there may be innate resistance via the rifampicin ADP-ribosyltransferase (Arr_Mab) mutation as it is a rifamycin-derived [60]. 

### 6.2. Bacteriophage Therapy

Bacteriophage treatment is a topic of growing interest in many multidrug-resistant infections, including MABC. Fluorescence microscopy demonstrated that phage can be uptaken into macrophages and lung epithelial cells as well as infect the intracellular MABC [61]. There has been success noted in case reports, including an engineered three-phage cocktail for a 15-year-old patient with cystic fibrosis and disseminated MABC following bilateral LT. The patient had clinical improvement with the resolution of infected skin nodules. Treatment was administered for 32 weeks, and it was well tolerated, except for diaphoresis and the feeling of being flushed for the first two days of therapy [62]. In another case report, a 26-year-old patient with cystic fibrosis and refractory MABC pulmonary infection was successfully treated with bacteriophage therapy, including two phages, allowing him to have a successful LT at day 379 of phage therapy [63]. Although the above successful case reports show promise as a potential option in treating refractory MABC cases in transplant patients, a major challenge is its ability to be scaled up to treat large numbers of patients as phage therapy needs to be individualized and requires a lot of time and resources.

## 7. Unique Therapeutic Considerations in SOT Patients

When selecting anti-mycobacterial therapy in SOT patients, their immunosuppressive agents must be taken into consideration, as there are important DDI. For macrolides, azithromycin less potently inhibits CYP450, which may increase CNI levels to a lesser degree when compared to clarithromycin and is likely an important factor in why the guidelines from the AST recommend azithromycin over clarithromycin for first-line treatment against MABC [9]. Furthermore, clarithromycin may also lead to increased mTOR inhibitor (i.e., sirolimus) levels by the same CYP450 inhibition, although this is not as readily seen with azithromycin. Rifamycins are potent inducers of CYP3A4 enzymes, and via this effect, they can markedly decrease the levels of the CNIs and sirolimus, precipitating organ rejection. MABC is intrinsically resistant to rifampin, so it is not recommended [64]. These points emphasize the importance of close clinical follow-up and interdisciplinary communication with members of the transplant team to ensure appropriate dosing of immunosuppressive agents to avoid adverse outcomes from DDI [7]. 

**Figure 1:**
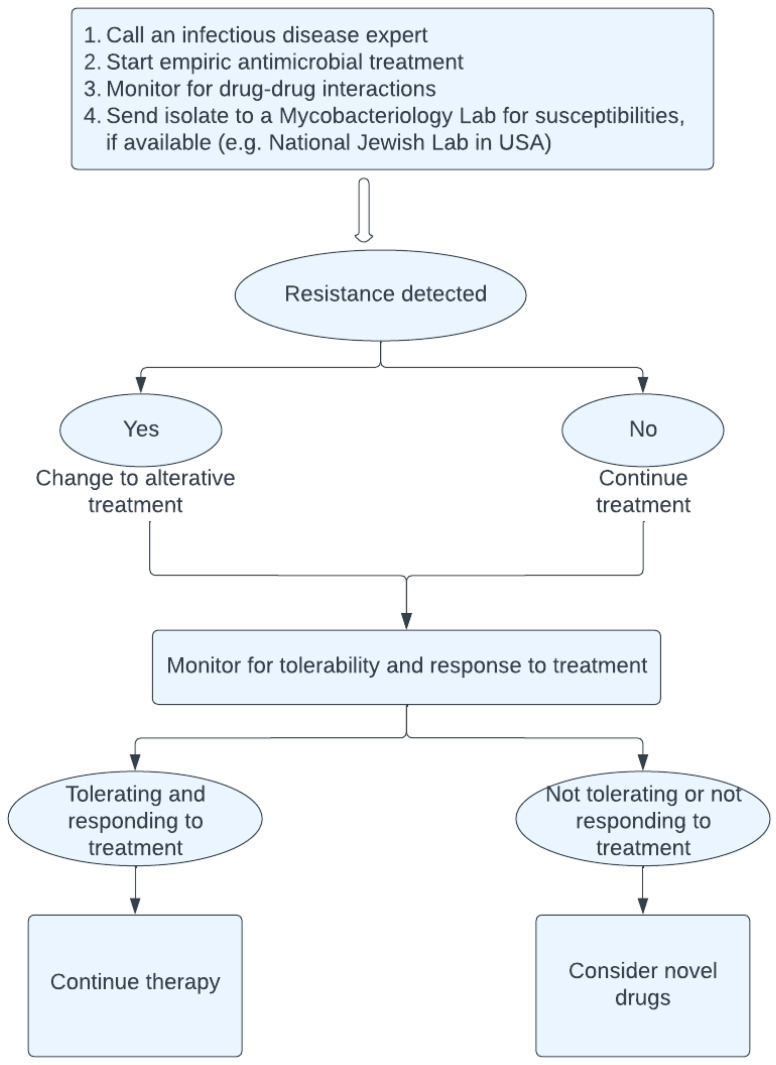
Management of *M. abscessus* in solid organ transplant patients.

The highlights for the management of MABC in SOT patients are illustrated in Figure 1. 

## 8. Summary

Managing MABC infections in SOT patients requires a meticulous approach, patience, and creativity to determine the correct combination of the limited therapeutic options given in vitro susceptibilities and tolerability. Although MABC diagnosis can be daunting to both patients and providers, we must utilize the expertise of our interdisciplinary teams and consider the potential use of novel therapies, especially in those patients who do not respond to or tolerate the standard drugs, to offer our patients the best chance of cure for this resilient organism.

## Figures and Tables

**Table 1:** Dosing of antimicrobials commonly used for *M. abscessus*.

Drug	Adult Dose	Renally Adjusted	Interacts with CNI or mTOR
Azithromycin PO or IV	250–500 mg daily	No	Yes
Clarithromycin PO	500 mg every 12 h	Yes	Yes
Amikacin IV ^1^	10–15 mg/Kg daily or 15–25 mg/Kg three times a week	Yes	No ^2^
Free amikacin (nebulized)	250–500 mg every 12 h	No	No
Liposomal amikacin (nebulized)	590 mg daily	No	No
Linezolid PO or IV	600 mg daily	No	No
Tigecycline IV ^3^	100 mg x1 (loading) followed by 50 mg every 12 h	No	No
Cefoxitin IV	8–12 g daily in divided doses	Yes	No
Imipenem IV	500 mg every 6 h or 1 g every 12 h	Yes	No
Clofazimine PO ^4^	100–200 mg daily	No	No

PO: by mouth; IV: intravenous; CNI: Calcineurin inhibitors; mTOR: mammalian target of rapamycin. ^1^ Data of other aminoglycosides were not included in this table. ^2^ The combination of amikacin IV with CNI or mTOR can potentiate renal toxicity. ^3^ Dosing of 50 mg daily is also acceptable. ^4^ Data of other novel therapies were not included in this table.
